# 

**Published:** 1818-02

**Authors:** 


### CATALOGUE OF MEDICAL BOOKS.

Chemical Amusement, &c. &c. The second Edition, with Plates, and considerably enlarged. By FREDERICK ACCUM, operative Chemist, &c. 12mo. boards, 9s.

The Dublin-Hospital Reports and Communications in Medicine and Surgery. Volume the First. 8vo. boards, 10s. 6d.

Dr. GORDON's Engravings of the Human Skeleton. 8vo.

On the Non-Contagious Nature of Yellow Fever; by Dr. VEITCH. 8vo.

---

### *NOTICES TO CORRESPONDENTS.*

*Several Original Communications (foreign and domestic) are arrived, and will appear in their turn.*

